# Hepadnaviral Lymphotropism and Its Relevance to HBV Persistence and Pathogenesis

**DOI:** 10.3389/fmicb.2021.695384

**Published:** 2021-08-06

**Authors:** Carla S. Coffin, Patricia M. Mulrooney-Cousins, Tomasz I. Michalak

**Affiliations:** ^1^Liver Unit, Department of Gastroenterology, Cumming School of Medicine, University of Calgary, Calgary, AB, Canada; ^2^Molecular Virology and Hepatology Research Group, Division of Basic Medical Sciences, Faculty of Medicine, Health Sciences Centre, Memorial University of Newfoundland, St. John’s, NL, Canada

**Keywords:** hepatitis B virus, woodchuck model of hepatitis B, lymphotyropism, virus genome integration, virus variants

## Abstract

Since the discovery of hepatitis B virus (HBV) over five decades ago, there have been many independent studies showing presence of HBV genomes in cells of the immune system. However, the nature of HBV lymphotropism and its significance with respect to HBV biology, persistence and the pathogenesis of liver and extrahepatic disorders remains underappreciated. This is in contrast to studies of other viral pathogens in which the capability to infect immune cells is an area of active investigation. Indeed, in some viral infections, lymphotropism may be essential, and even a primary mechanism of viral persistence, and a major contributor to disease pathogenesis. Nevertheless, there are advances in understanding of HBV lymphotropism in recent years due to cumulative evidence showing that: (i) lymphoid cells are a reservoir of replicating HBV, (ii) are a site of HBV-host DNA integration and (iii) virus genomic diversification leading to pathogenic variants, and (iv) they play a role in HBV resistance to antiviral therapy and (v) likely contribute to reactivation of hepatitis B. Further support for HBV lymphotropic nature is provided by studies in a model infection with the closely related woodchuck hepatitis virus (WHV) naturally infecting susceptible marmots. This animal model faithfully reproduces many aspects of HBV biology, including its replication scheme, tissue tropism, and induction of both symptomatic and silent infections, immunological processes accompanying infection, and progressing liver disease culminating in hepatocellular carcinoma. The most robust evidence came from the ability of WHV to establish persistent infection of the immune system that may not engage the liver when small quantities of virus are experimentally administered or naturally transmitted into virus-naïve animals. Although the concept of HBV lymphotropism is not new, it remains controversial and not accepted by conventional HBV researchers. This review summarizes research advances on HBV and hepadnaviral lymphotropism including the role of immune cells infection in viral persistence and the pathogenesis of HBV-induced liver and extrahepatic diseases. Finally, we discuss the role of immune cells in HBV diagnosis and assessment of antiviral therapy efficacy.

## Introduction

Hepatitis B Virus (HBV) is known as a hepatotropic virus that can cause life-threatening liver diseases, such as chronic hepatitis B (CHB), cirrhosis and hepatocellular carcinoma (HCC). Early studies revealed many exciting facets of the HBV biology including its ability to infect and persist in cells of the immune system; an entity that was subsequently termed hepadnaviral lymphotropism. However research on this subject has largely subsided. Identification of the nature of this event and its significance to HBV biology, course of infection and pathogenesis of liver and extrahepatic disorders received limited attention and is currently underappreciated by many HBV researchers. This is in sharp contrast to studies on other non-cytopathic, long-term persisting viral pathogens in which the ability to infect immune cells were actively investigated. In fact, lymphotropism was found to be essential to the persistence and to the pathogenesis of disease induced by many viruses. There are several examples of these viruses, including human immunodeficiency virus type 1 (HIV), hepatitis C virus (HCV), Epstein B virus, cytomegalovirus, measles virus, and a classical model of persistent lymphotropic infection with lymphocytic choriomeningitis virus (Klenerman and Zinkernagel, 1997; Ciurea et al., 1999; Sevilla et al., 2000; Sundaravaradan et al., 2006; Pham et al., 2008; Taylor et al., 2015; Leng et al., 2017; Griffin et al., 2018; Michalak, 2018; Farrell et al., 2019). Nevertheless, the understanding of HBV lymphotropism has advanced and HBV lymphotropism has gained more validity in recent years. Valuable support was provided by investigations in a model HBV infection system comprised of the closely related woodchuck hepatitis virus (WHV) infecting naturally susceptible marmots. This infection model reproduces with high accuracy many aspects of HBV biology, including replication scheme, tissue tropism, immunological processes accompanying infection, as well as induction of symptomatic and silent infections and a progressive liver disease resulting in chronic WHV hepatitis (CWH) advancing to HCC (Popper et al., 1981; Michalak, 2000; Menne and Cote, 2007; Roggendorf et al., 2015; Michalak, 2020).

**FIGURE 1:**
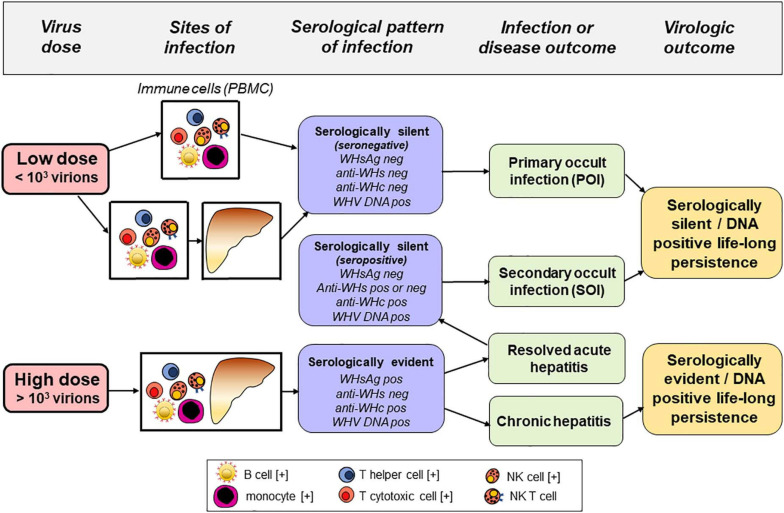
Sites of infection, serological (immunovirological) and molecular (DNA) profiles, and outcomes of hepadnaviral infection in the woodchuck model of hepatitis B after intravenous inoculation with very low (liver non-pathogenic) or high (liver pathogenic) dose of WHV. Cell types found infected with HBV and/or carrying HBV DNA integration in patients with chronic hepatitis B are marked with [+].

To facilitate better appreciation of advantages of the woodchuck-WHV model, a brief comparison of the natural course of HBV and WHV infections and the profiles of serological (immunovirological) and molecular infection markers are summarized. In humans, resolution of acute HBV infection occurs after HBV surface antigen (HBsAg) clearance and development of anti-HBsAg antibodies (anti-HBs). Antibodies to HBV core antigen (anti-HBc) develop in the pre-acute phase of infection and remain throughout the lifespan. Similarly, symptomatic WHV infection in woodchucks begins as acute WHV hepatitis that is serum WHV surface antigen (WHsAg) positive that spontaneously resolves in the majority of adult animals with development of antibodies to WHsAg (anti-WHs). This self-limited acute hepatitis is followed by essentially asymptomatic, life-long virus persistence termed as secondary occult infection (SOI) or seropositive-occult HBV infection (OBI) (Figure 1; Michalak et al., 1999; Raimondo et al., 2008; Michalak, 2020). SOI is serum WHsAg-negative, but antibodies to WHV core antigen (anti-WHc) (equivalent to anti-HBc) persist lifelong and anti-WHs (equivalent to anti-HBs) may be present or slowly decline to undetectable levels. After resolution of acute hepatitis, the liver intermittently shows minimal to moderate inflammation with periods of normal or nearly normal morphology. However, HCC develops in about one fifth of woodchucks with SOI. Levels of serum WHV DNA decline ∼ 5–6 logs after resolution of acute hepatitis (i.e., from 10^3^–10^6^ copies/mL or virus genome equivalents, vge, to <100–200 vge/mL). Molecular indicators of WHV replication, virus mRNA and covalently closed circular DNA (cccDNA) are detectable in the liver and cells of the immune system which, among others sites, include peripheral blood mononuclear cells (PBMC) (Michalak et al., 1999; Mulrooney-Cousins and Michalak, 2015). Similar to adult HBV infection, a minority (10–15%) of WHV-infected animals with acute hepatitis progress to chronic hepatitis that is serum WHsAg and anti-WHc reactive along with viral loads up to 10^8^–10^10^ vge/mL. Chronic woodchuck hepatitis is characterized by prolonged liver necro-inflammation with predominantly lymphocytic infiltrations and histological features that parallel CHB in humans (Popper et al., 1981; Michalak, 1998; Tennant and Gerin, 2001). Chronic woodchuck hepatitis advances to HCC in up to 90% of woodchucks compared to only 5% HCC rate in humans with CHB (Tennant and Gerin, 2001; Tennant et al., 2004; Yang et al., 2019). In addition, there is a form of serologically silent, but molecularly evident infection accompanied by WHV-specific T cell responses but not by virus-specific humoral immunity (Michalak et al., 2004; Gujar and Michalak, 2009). This infection is caused by WHV quantities equal to or lower than 10^3^ virions and, in contrast to SOI, does not protect from re-infection with WHV. This form has been designated as primary occult infection (POI) (Figure 1; Michalak et al., 2004; Mulrooney-Cousins and Michalak, 2015), also termed as seronegative-OBI in humans (Raimondo et al., 2008). Despite persistence of low level serum WHV DNA (<100 vge/mL), serum WHsAg, anti-WHc and anti-WHs remain negative in POI. Virus genome and its replication are only detectable in PBMC and organs of the immune system. Over time, WHV genomes are eventually detected in the liver that supports low-level WHV replication in the context of normal liver biochemistry and histology (Mulrooney-Cousins et al., 2014). Nonetheless, infectious and liver pathogenic WHV is produced during the animal’s entire lifespan and HCC develops in about 20% of animals with POI (Mulrooney-Cousins et al., 2014). Although unproven, this form of infection could be responsible for cryptogenic HCC in patients tested negative for HBV by standard clinical laboratory tests (Wong et al., 2011; Mak et al., 2020).

In this review, we summarize the advances made over the last five decades in HBV research and hepadnaviral lymphotropism. We also discuss the role of immune cells’ infection in HBV persistence, the pathogenesis of liver and immunoproliferative diseases, and extrahepatic assessment for hepatitis B diagnosis and treatment.

## Early Studies on HBV Lymphotropism

Early studies after the discovery of HBV suggested that the virus might reside and propagate in cells other than hepatocytes, particularly in those of the immune system. However, evidence was limited mainly due to limitations in the methods identifying HBV genome and its replicative intermediates at that time. They were predominantly based on nucleic acid hybridization (NAH) with the lowest limits of detection only 10^5^–10^7^ virus genome copies or vge/mL (Michalak et al., 1994; Liu and Yao, 2015; Lau et al., 2020). Other limitations were related to immunodetection of viral proteins and imprecise differentiation between intracellular and the cell surface-associated proteins. Nonetheless, there was a number of compelling studies which demonstrated HBV infection of bone marrow cells from patients with CHB by demonstrating *via* immunoelectron microscopy intracellularly located HBV envelope and nucleocapsid (core) proteins (Romet-Lemonne et al., 1983; Elfassi et al., 1984). These cells also produced viral particles with buoyant density and ultrastructural features of HBV virions [i.e., Dane particles (Dane et al., 1970)] and released virus envelope particles with a diameter of 22 nm (Romet-Lemonne et al., 1983; Elfassi et al., 1984). Other works showed susceptibility of normal bone marrow cells to HBV *in vitro* and implied that virus can infect different lineages of hematopoietic stem cells and suppress bone marrow hematopoiesis (Zeldis et al., 1986; Steinberg et al., 1990). Early studies also indicated that examination of easily accessible PBMC, composed of lymphocytes and monocytes, allows for HBV DNA detection in the majority of symptomatically infected individuals. In addition, low molecular weights of HBV DNA, suggesting replicative intermediates, and viral mRNA, implying active virus transcription, were detected (Pontisso et al., 1984; Laure et al., 1985; Noonan et al., 1986; Pasquinelli et al., 1986). Different subpopulations of PBMC, such as B and T lymphocytes and monocytes, were found to be infected by HBV (Korba et al., 1986; Noonan et al., 1986; Yoffe et al., 1986; Lauré et al., 1987; Figure 1). HBV nucleic acid sequences were also found in autopsy spleen, lymph nodes, and at other locations in acutely and chronically infected individuals when blots of cellular DNA or RNA where tested with HBV-specific probes by Southern or Northern blot NAH (Dejean et al., 1984; Yoffe et al., 1990; Zoulim et al., 1991; Mason et al., 1993). Nonetheless, no histologically apparent changes were found at the extrahepatic sites of HBV detection (Yoffe et al., 1990).

In the search for HBV proteins mediating virus binding to immune cells, recombinant particles containing recombinant preS1 (large), preS2 (middle), or S (small or major) envelope proteins were constructed (Pontisso et al., 1991). The N-terminal portion of the preS1 protein, encompassing amino acids in positions 27–49, was identified as the HBV cell-binding site. This site specifically recognized hepatocytes and PBMC-derived B cells, monocytes and, to a lesser degree, T cells. The same preS1 sequence was identified in the preceding studies to bind human hepatocyte-like HepG2 cells, B lymphocytes, hematopoietic B cell lines, and to some other cell types (Neurath et al., 1986, 1990). Nevertheless, the precise mechanism of HBV entry to immune cells remains unknown. The sodium taurocholate co-transporting polypeptide (NTCP) receptor, which mediates HBV entry to human hepatocytes (Yan et al., 2012), is unlikely to be involved. The data from one of our laboratories showed that the transcription levels of NTCP in human PBMC and woodchuck PBMC, spleen and bone marrow were very low (<200 copies/μg total RNA) compared to HBV-susceptible human HepaRG cells (>1 × 10^6^ copies/μg total RNA). However, the usage of distinct receptors to infect different cell types is well known among viruses (Klatzmann et al., 1984; Nemerow et al., 1986; Sakaguchi et al., 1986; Jin et al., 1994; Kasai et al., 1994; Zaitseva et al., 1997; Tatsuo et al., 2000; Sarhan et al., 2012).

**FIGURE 2:**
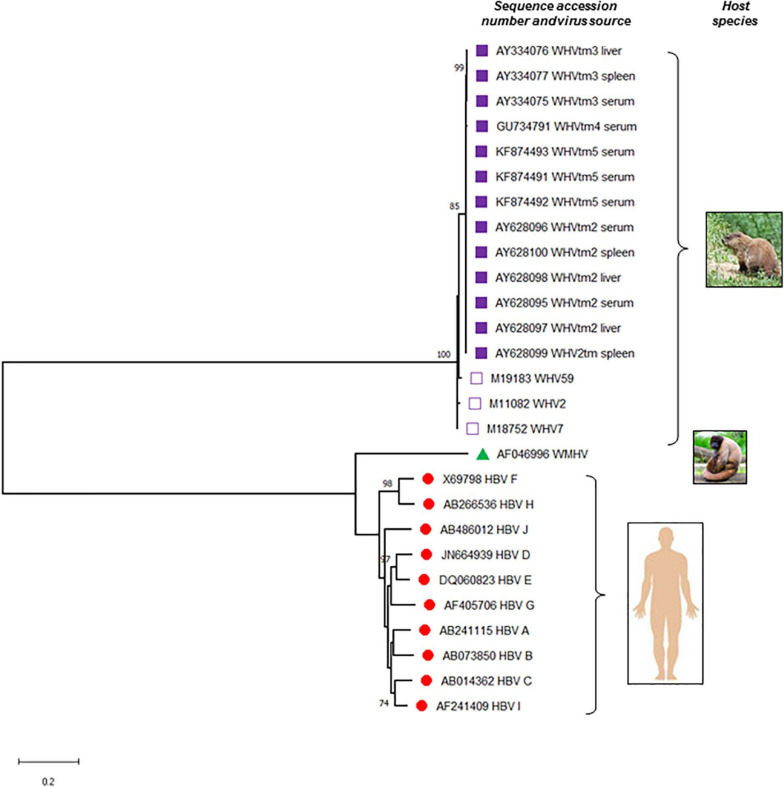
Phylogenetic tree of the nucleotide sequences illustrating the evolutionary relationship between hepatitis B virus (HBV) and woodchuck hepatitis virus (WHV). Complete sequences of all 10 known HBV genotypes (A to J), woodchuck hepatitis virus (WHV) identified at different locations of the virus natural occurrence, and a representative sequence of wooly monkey hepatitis virus (WMHV) were included. Full genomic sequences of WHVtm2, WHVtm3 WHVtm4, and WHVtm5 were obtained after clonal sequencing, while those of WHV2, WHV7, and WHV59 by traditional Sanger sequencing. If the source material of virus was not listed, serum or plasma was used to isolate viral DNA sequenced. The sequences and their accession numbers obtained from GenBank (National Center for Biotechnology Information, National Library of Medicine, Bethesda, MD, United States) are indicated. Sequences were aligned using MAFFT and the trees were generated using MEGA software version X (www.megasoftware.net). For the analysis, the Maximum Likelihood method and General Time Reversible (GTR) model with Gamma distribution (+*G*, parameter = 0.6290) were used. The tree with the highest log likelihood (–17303.75) is shown. Only bootstrap values of above 70% are shown and distance scale bare is included. Gaps and missing data were eliminated leaving of 2968 positions in the dataset.

Early studies in animal models of HBV infection, such as Pekin ducks, woodchucks and chimpanzees, uncovered hepadnaviral nucleic acid sequences and proteins at extrahepatic locations, most often in PBMC, spleen, pancreas, and kidneys (Halpern et al., 1983; Korba et al., 1986; Jilbert et al., 1987; Lieberman et al., 1987; Korba et al., 1988b; Ogston et al., 1989; Hosoda et al., 1990; Walter et al., 1991; Figure 2). Hence, hepadnaviral DNA and RNA were detected in PBMC of HBV-infected chimpanzees and WHV-infected woodchucks, and in B and T cells of chimps when probed for virus-specific signals by NAH *via* Southern and Northern blot analyses (Korba et al., 1986). Replicative forms of the WHV genome were also documented in lymphoid cells from spleens (splenocytes) of woodchucks with chronic hepatitis, while animals convalescent from acute hepatitis were rarely reactive by classical NAH methods (Korba et al., 1987). In addition, extracts from splenocytes subjected to buoyant density gradient centrifugation and examination of the resulting fractions by electron microscopy showed 27-nm diameter WHV core particles carrying virus RNA-DNA hybrids and activity of endogenous DNA polymerase (Korba et al., 1987). These findings clearly showed that woodchuck splenocytes, which predominantly contain lymphocytes when purified, were the site of WHV replication. They also suggested that these cells could be the site of virion assembly, although whether they produced infectious virus was unknown at that time. In a related study, it was shown that WHV replication could be augmented in WHV DNA-positive PBMC after their *ex vivo* exposure to a bacterial mitogen, lipopolysaccharide (LPS) (Korba et al., 1988a). Following stimulation, WHV core particles carrying virus DNA replicative intermediates and endogenous DNA polymerase were found within the cells and the mature virions in the cell culture supernatant. This study showed that activation of cell proliferation coincides with reactivation of a latent WHV infection in circulating immune cells and that this may result in virion production.

Further, a WHV site capable of strict host and cell-type specific recognition was identified in the extreme N-terminal portion of the virus preS1 protein (Jin et al., 1996). The crucial domain of the site was mapped to residues 10–13 (NPDK motif). Notably, a similar motif exists within the HBV preS1 24–47 amino acid sequence at positions 40–43 and is comprised by residues NPDW. Synthetic analogs of the WHV cell-binding site bound woodchuck splenocytes, PBMC and hepatocytes in a species-restricted manner with characteristics of a specific ligand-receptor interaction. These peptides interacted at ∼1000 greater fold affinity with splenocytes and PBMC compared to interaction with primary woodchuck hepatocytes. Moreover, proteolytic cleavage and, to a lesser degree, low pH were required to present the binding activity on WHV envelope, suggesting that natural folding of the protein may protect the site and hinder the access to the site by specific antibodies (Jin et al., 1996). These findings closely resemble the recent data showing that HBV binding to human hepatocyte requires preS1 protein modification and this happens by translocating preS1 protein across the envelope onto virus surface (Seitz et al., 2016). In addition, the binding of WHV to hepatocyte and splenocyte plasma (surface) membranes involves a 330-kD proteoglycan constituted by the protein core and O-linked heparan sulfate and N-linked polymannose (DeSousa, 1994). The binding of WHV to the 330-kD receptor and woodchuck hepatocyte plasma membranes is inhibited by heparin. This effect is comparable to that of heparin on the binding of HBV to human hepatocytes (Sureau and Salisse, 2013).

Early studies identified, with a high degree of confidence, that HBV infects both circulating and lymphoid organ residing immune cells. They suggested that these cells are a site of active HBV replication and might produce infectious virus under certain circumstances. The findings in the woodchuck infection model strongly supported these possibilities and the essentially lymphotropic nature of WHV. Other HBV animal models were less extensively studied but still provided insight on hepadnaviral lymphotropism (Figure 2).

## Identification of Hepadnaviral Lymphotropism in the ERA of Nucleic Acid Amplification Methods

The advent of polymerase chain reaction (PCR) enabled development of sensitive and standardized methods for detection and quantitation of hepadnavirus genomes and replication intermediates, including mRNA and cccDNA (Baginski et al., 1991; Stoll-Becker et al., 1997). The new research tests facilitated identification of viral DNA and RNA with sensitivity < 10 vge per mL or per microgram (μg) of total cellular DNA or RNA, and cccDNA < 100 copies/μg of total cellular DNA (Michalak et al., 1999; Lew and Michalak, 2001; Michalak et al., 2004). The combination of PCR and NAH of amplicons increased sensitivity of signal detection, confirmed specificity, and strengthened validity of negative controls and quantification standards (Michalak et al., 1994, 1999, 2004; Coffin and Michalak, 1999; Coffin et al., 2011a; Mulrooney-Cousins et al., 2014). In some cases with very low viral loads, detection of virus-specific PCR signals was only possible by the addition of NAH analysis. Further, NAH is well justified considering the very high sensitivity of research assays and the risk of accidental contamination, especially with nested PCR. For the same reasons, rigorous precautions are routinely applied during all steps of viral detection, i.e., from the collection and processing of samples, throughout DNA or RNA extraction, to reagent preparation and assay execution. Routine standard controls should include a “mock” sample that is processed in parallel throughout all steps with the test samples and water instead of test DNA or cDNA during amplification. To confirm specificity, controls consisting of nucleic acid preparations of the same type of sample from a known positive or negative virus sample are used. Primer sets specific for different hepadnaviral genes are normally utilized to ascertain detection. Additionally, direct Sanger sequencing of each amplicon may be used to confirm specificity. PCR with plasmids carrying hepadnaviral sequences should not be done at the same time as test samples to mitigate cross-contamination. For identification of viral mRNA by PCR with the reverse transcription (RT) step (i.e., RT-PCR), test RNA should be transcribed both with and without (control) of RT enzyme to exclude contamination with virus DNA. Alternatively, test RNA should be pre-treated with DNase before PCR, although this may deplete the RNA template.

To augment detection and/or diagnosis of hepadnaviral replication in immune cells, various specialized assays to enrich the virus template along with sensitive quantitative PCR have been developed. These approaches include: (1) Stimulation of circulating or lymphatic organ-derived immune cells *ex vivo* with cell stimulating mitogens (Korba et al., 1988a; Michalak et al., 1999; Lew and Michalak, 2001; Lau et al., 2020). Mitogen stimulation is similar to other methods used to enhance virus levels in immune cells (Hyypiä et al., 1985; Gowda et al., 1989; Frenkel et al., 1990; Joshi et al., 2004; Pham et al., 2005; Pham et al., 2008); (2) Direct or clonal sequencing of the PCR amplified viral genes or the complete virus genome to identify compartmentalization of viral variants (Günther et al., 1995; Rokuhara et al., 2000; Lee et al., 2015); (3) Enumeration of virally infected immune cells (Mulrooney and Michalak, 2003), and (4) Detection of virus-host genome integration (Mulrooney-Cousins et al., 2014; Lau et al., 2020). These studies have meaningfully advanced recognition of the nature, biological characteristics and potential pathological consequences of hepadnaviral lymphotropism. They also facilitated systematic research using the woodchuck model of HBV infection, which is the primary information source regarding the attributes of HBV lymphotropism. Similar studies in HBV infected patients are limited by the acquisition of suitable investigative material, especially in the initial stages of infection. In current clinical practice, PCR-based approaches are only used for detecting and quantifying HBV DNA, identifying HBV genotypes and selected drug resistant variants in serum or plasma from patients (Mulrooney-Cousins and Michalak, 2017; Coffin et al., 2019).

The first indication of hepadnavirus lymphotropism is usually detection of virus DNA in PBMC, lymphoid organs and their immune cells isolated from naturally infected hosts or immune cells after *in vitro* exposure to virus. However, the signal may originate from viral particles or virus free DNA fragments adhered to the cell surface or tissue from contaminating blood or culture medium. Therefore, removal of these potential contaminants is done by limited digestion of the cell surface with trypsin and DNase followed by extensive washing prior to extraction of nucleic acid, as reported (Michalak et al., 1994; Lew and Michalak, 2001; Lau et al., 2020). It is also important to test the final cell wash for viral DNA and, if negative, this is a strong indicator that cell-derived DNA is of intracellular origin. However, intracellular detection of hepadnaviral RNA transcripts and cccDNA (in either hepatocytes or immune cells) are the most valued and direct indicators of replication. A strong sign of active replication in the immune cell compartment is detection of unique virus variants when comparing to virus sequences identified in serum and liver from the same individual or animal (Rokuhara et al., 2000; Lau et al., 2020). Virus propagation in immune cells is also indicated by the appearance of virus proteins in *de novo* infected cells or detection of intracellular proteins in naturally infected cells *ex vivo*. However, this finding needs to be interpreted in the context of the specific immune cell type involved, since some phagocytic cells (i.e., monocytes, macrophages) and to a lesser degree B cells may passively carry phagocytosed virus. The ultimate confirmation of hepadnaviral lymphotropism is demonstration of the infected immune cells capability to produce infectious virus that is able to establish productive replication in susceptible cells *in vitro* or *in vivo* using a relevant animal model.

## HBV Infection of Hematopoietic and Mesenchymal Stem Cells

As noted, early studies using bone marrow from HBV-infected individuals showed that HBV can replicate and assemble virions in bone marrow cells, and healthy hematopoietic stem cells were susceptible to HBV infection. Since then, a number of studies have investigated HBV tropism toward both hematopoietic and mesenchymal stem cells (Romet-Lemonne et al., 1983; Elfassi et al., 1984; Zeldis et al., 1986). In this regard, the susceptibility of human pluripotential CD34+ hematopoietic stem cells, i.e., precursors of immune cells, myeloid and erythroid cell linages, to HBV infection was investigated (Shi et al., 2014). HBV mRNA was found in cultured stem cells from healthy volunteers following exposure to HBV-positive sera, with progressive increases in HBV DNA levels and in intracellular HBsAg expression. This work also suggested that CD34+ peripheral blood stem cells (a minor PBMC subset), could be infected with HBV since virus DNA levels gradually increased in the cell extracts and the cell supernatants after several days of culture (Huang et al., 2016). Another study showed HBV mRNA and HBV DNA, and integration of HBV DNA into chromosomes of CD34+ hematopoietic stem cells from bone marrows of CHB patients (Ma et al., 2011). The data also suggested that HBV-infected hematopoietic stem cells might generate T cells with defective proliferation and production of cytokines, such as interleukin-2 (IL-2) and interferon gamma.

Hepatitis B virus replication was also evident in human bone marrow mesenchymal stem cells after exposure to serum with a high HBV load (Ma et al., 2011). The bone marrow cells were negative for CD34, a marker of hematopoietic stem cells (Sidney et al., 2014), but reactive for CD105 and CD90. CD105+ and CD90+ mesenchymal stem cells are also hepatocyte progenitors (Aurich et al., 2007). Indicators of HBV replication included the progressive increase in cell-associated HBV DNA levels and release of HBsAg and HBV e antigen, and detection of HBV core protein and cccDNA. Another study found that differentiated umbilical cord matrix stem cells are prone to HBV infection and can support the complete viral life cycle. This conclusion was based on detection of virus RNA, *de novo* synthesis of HBV proteins, a dose-dependent inhibition of virus replication by an antiviral drug, tenofovir disoproxil fumurate, and by secretion of infectious virus (Paganelli et al., 2013).

The ability of HBV to infect hematopoietic cell precursors which can differentiate to the entire spectrum of immune cells, including lymphocytes and monocytes, further attests to the lymphotropic nature of HBV. This may also explain why different immune cell subsets could be simultaneously infected in the same individual. HBV ability to infect these cells likely plays an important role in viral persistence. Virus propagation within the immune privileged compartment, protected from immune surveillance, may directly affect immune cell function and promote its persistence.

## HBV Incidence and Replication in Differentiated Immune Cell Subsets

**FIGURE 3:**
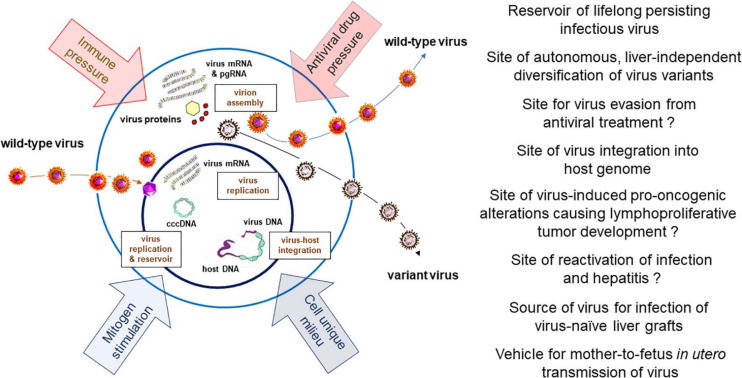
Schematic presentation of HBV and WHV infection markers which can be detected in infected immune cells, factors influencing hepadnavirus replication, and documented or expected (marked with question mark) biological and pathogenic consequences of virus residing in or produced by immune cells.

The ability of HBV to infect immune cell progenitors offers a plausible explanation why virus genome and its replication are detectable throughout the entire immune system, including cells residing in lymphatic organs and those settled within loose lymphatic tissue. Studies applying PCR-based methods generated a wealth of data that overall strongly supported the existence of productive replication of HBV and WHV in the immune cell compartment (Figure 3). Among others, the presence of HBV mRNAs and cccDNA was documented in naturally circulating PBMC without *ex vivo* stimulation with mitogens or viral antigenic epitopes isolated from highly viremic patients (Stoll-Becker et al., 1997). A PCR protocol allowing distinction of the different HBV mRNAs for the virus large (preS1) and small (S) envelope proteins and the X protein, and the nested PCR discriminating HBV cccDNA from HBV relaxed circular DNA (rcDNA) were established for the purpose of that study. In addition to total PBMC, B and T cell subsets were analyzed for HBV mRNAs. Both cell subsets carried HBV mRNA, but B cells were reactive at higher levels (Stoll-Becker et al., 1997). In another study, HBV DNA and mRNA in PBMC cell subsets from patients with CHB were examined. The HBV genome was detected in immune cells positive for CD3, CD4, CD19, and CD56, whereas virus RNA was identified in cells reactive for CD19 (B cells) and CD56 (NK cells) only (Chemin et al., 1994). In the same study, HBcAg was detected in B and NK cells, and the same cell types were reactive for HBsAg at the highest ratios. The study authors concluded that HBV replication occurred in B and NK cells in the cases investigated. HBV infection rates and viral titers were also assessed in subpopulations of PBMC from patients with acute or chronic hepatitis B using limiting dilution nested PCR (Trippler et al., 1999). The highest virus DNA loads were detected in monocytes and B cells, followed by CD8+ T cells, NK cells and CD4+ T cells. The data suggested HBV replication or selective uptake within specific cell subsets was significantly more pronounced in chronic compared to acute HBV infection.

In a more recent study, PBMC and their subsets, i.e., CD4+ and CD8+ T cells, CD19+ B cells, CD14+ monocytes, and CD56+ NK cells, from individuals infected with HBV alone or co-infected with HIV, either treatment naïve or treated, were examined for HBV DNA and replicative intermediates by PCR/NAH assays (Lee et al., 2015). Total PBMC and their cell subsets were also tested for drug-resistant HBV variants by deep sequencing. HBV DNA, mRNA, and cccDNA were detected within all PBMC from both patients’ subgroups investigated. There were no significant differences in the frequency of HBV DNA detection in total PBMC and their subsets from treatment naïve and nucleoside/tide analogs (NA)-treated patients mono-infected with HBV. HBV cccDNA copy numbers were not different in PBMC from untreated and treated individuals infected with HBV alone. In cell subsets from PBMC of the HBV–HIV co-infected patients, at least one and as many as four subsets were HBV DNA reactive, except CD4+ T cells which were uniformly negative. HBV cccDNA was detected in CD8+ T cells and NK cells from these patients. Finally, mutations at nucleotides associated with drug resistance or immune escape were identified in the CD4+ and CD56+ cells from two HBV mono-infected cases on NA therapy. Overall, this study and other studies by the same group (Coffin et al., 2014) demonstrated that in HIV co-infection, HBV remained detectable in total PBMC and in their subsets despite potent suppressive antiviral therapy that was associated with a significant decrease in the HBV load in plasma. The findings confirmed that different immune cell types support HBV replication (Figure 1). They also suggested that HIV infection could affect HBV lymphotropism, as implied by the absence of HBV in CD4+ T cells from HBV-HIV co-infected individuals.

## Infectivity and Pathogenicity of WHV Residing in the Immune Cell Compartment

Woodchuck hepatitis virus replication within immune cells, and infectivity and liver pathogenic competence of the virus produced by these cells were extensively investigated in by *in vitro* and *in vivo* conditions. Hence, WHV-infected splenocytes, containing predominantly lymphocytes, and PBMC from animals with chronic hepatitis, which were depleted of potentially cell surface-adhered WHV virions and WHV DNA fragments by enzymatic treatment, served as a source of virus to infect woodchuck hepatocytes [i.e., WCM-260 cells (Diao et al., 1998; Guy et al., 2006)] or WHV-naïve PBMC in co-cultures (Lew and Michalak, 2001). The productive replication in naturally and *de novo* infected cells was verified by: (1) Detection of WHV-specific DNA and cccDNA; (2) Transmission of infection to virus-naïve hepatocytes and PBMC by WHV secreted by naturally infected splenocytes or PBMC; (3) Infection of healthy woodchucks with WHV produced by naturally infected splenocytes, and (4) Infection of healthy woodchucks with WHV recovered after passage in hepatocytes that were initially exposed to WHV released from naturally infected splenocytes or PBMC. In woodchucks, WHV produced by naturally infected splenocytes established classical acute hepatitis (for further details see page 8), while WHV passaged in hepatocytes induced an asymptomatic, seronegative but WHV DNA-positive infection. We have since learned that such pattern of serum WHsAg and anti-WHc negative but WHV DNA reactive infection reflects POI and that the variable infection outcomes seen in this study were due to different amounts of virus administered (Michalak et al., 2004; Mulrooney-Cousins et al., 2014). Overall, the results showed that immune cells, either circulating or residing in the lymphoid organ, support productive replication of WHV. This virus is infectious to hepatocytes as well as immune cells both *in vitro* and after administration to susceptible woodchucks and can cause hepatitis.

To enumerate WHV DNA-positive PBMC, WHV DNA has been amplified within intact immune cells by *in situ* PCR coupled with flow cytometric identification of cells carrying amplified virus genome (Mulrooney and Michalak, 2003). This method can specifically count infected cells in different forms of WHV infection and disease stages, including in serial samples from animals at baseline (healthy, uninfected state) throughout hepatitis to recovery or progression to chronic hepatitis and HCC. To ensure that the virus DNA was located intracellularly, DNase-trypsin-DNase treatment to strip potentially attached virions or residual virus DNA on the cell surface was performed prior to *in situ* PCR. The results showed that a significant proportion of immune cells comprising PBMC were WHV DNA reactive in symptomatic, serum WHsAg-positive animals with acute hepatitis or chronic hepatitis (mean 9.6%, range 3.4–20.4%). PBMC collected from animals with secondary occult and primary occult infection were also WHV genome reactive, but at a significantly lower frequency (mean 4.4%, range 1.1–14.6%) (Mulrooney and Michalak, 2003). The detection of WHV cccDNA in immune cells positive by *in situ* PCR/flow cytometry confirmed virus replication in these cells. This study provided a unique approach to quantify cells with intracellularly located hepadnaviral DNA and suggested that a similar strategy can be developed to enumerate infected immune cell subsets and cells with molecular replication markers. A comparable approach has been used to enumerate PBMC infected by other lymphotropic viruses, such as HIV or HCV (Re et al., 1994; Muratori et al., 1996). The number of HIV-positive cells ranged from 0.6 to 20% (Re et al., 1994), while HCV-specific signals were detected in 0.2–8.1% of PBMC, frequently in the absence of clinical signs of HCV infection (Muratori et al., 1996). Overall, these data indicate that WHV ability to infect immune cells is comparable to the properties of other lymphotropic viruses.

A number of studies provide evidence in support of a pathogenic competence of WHV produced within the immune cell compartment by investigating biological properties of WHV persisting as secondary occult or primary occult infection. The most relevant findings are as follows. In one of the study, PBMC were collected from two animals at more than a year after resolution of acute hepatitis to investigate the longevity of WHV persistence. The cells were cultured in the presence of LPS and their culture supernatant *i.v.* administered to two naïve woodchucks (Michalak et al., 1999). One of the animals developed an episode of serum WHsAg-positive, histologically evident acute hepatitis, and acquired transiently anti-WHs and anti-WHc that lasted up to the end of the 42-month observation period. WHV DNA and mRNA were detected after resolving acute hepatitis in the majority of PBMC samples at levels between 0.05 and 5 vge/10^4^ cells and in all liver biopsies collected at about 12-month intervals at 0.2 to 2 vge/10^4^ cells. The second animal established typical serum WHsAg-reactive, histologically confirmed chronic hepatitis that advanced to HCC within 25 months after inoculation with PBMC-derived virus. High levels of WHV DNA in PBMC (5 × 10^3^ – 5 × 10^4^/10^4^ cells) and in liver biopsies (>2 × 10^6^ vge/10^4^ cells) were detected throughout the animal follow-up. In addition to WHV DNA and mRNA, full length WHV DNA was identified in liver biopsies from the first animal and in autopsy serum and hepatic and splenic tissues from the second woodchuck using PCR with back-to-back primers located at the WHV nick region (Michalak et al., 1999). In another experiment, PBMC obtained at more than 4.5 years after resolution of acute hepatitis were enzymatically treated to eliminate possibly adhered WHV virions and DNA, and injected *i.v.* at more than 80% viability to a WHV-naïve woodchuck. The animal developed infection in which WHV DNA appeared in serum and PBMC, while liver samples collected at 2 and 6 months post-infection (p.i.) and at autopsy were negative along with normal histology. Serum WHsAg and anti-WHc were not detected, whereas WHV DNA and mRNA were found in PBMC, bone marrow and pancreas at autopsy. Thus, although the animal acquired WHV infection, the liver was uninfected. In subsequent studies, we appreciated that this pattern reflected persistent primary occult infection or POI (Michalak et al., 2004). This study provided conclusive evidence that immune cells are a lifelong reservoir of WHV and they can produce infectious, liver pathogenic virus capable of inducing hepatitis that retains oncogenic potency, indicated by HCC development (Michalak et al., 1999).

In another study already mentioned (Lew and Michalak, 2001), WHV produced by cultured splenic lymphocytes from an animal with chronic hepatitis, which were enzymatically pretreated to eliminate possibility of carry-over of an extracellular virus, were injected *i.v.* at 4.8 × 10^6^ virions to a healthy woodchuck. The animal developed serum WHV surface antigen and WHV core antibody reactive infection along with WHV DNA in serum, liver and PBMC, and histologically evident acute hepatitis. This was followed by hepatitis resolution and hepatic WHV loads < 100 vge/10^4^ cells at 6 and 9 months p.i. anti-WHs antibody were not detectable, while anti-WHc antibody persisted to the end of follow-up. Hence, woodchucks inoculated with WHV produced by naturally infected, unstimulated splenocytes, developed classical acute hepatitis that resolved, yet infection continued as secondary occult infection. This data also showed that WHV derived from immune cells is infectious, can induce liver injury and establish a persistent infection.

## Hepadnavirus Infection of the Immune System Without Engagement of the Liver

The authenticity of hepadnaviral lymphotropism was definitively established based on studies of naturally occurring and experimentally induced asymptomatic, serologically silent WHV infection. This is now recognized as a distinct form of occult infection, designated as primary occult infection (Figure 1). This form of infection was first discovered in the offspring of woodchuck mothers with resolved acute hepatitis, even though most animals developed otherwise protective anti-WHs (Coffin and Michalak, 1999). The offspring carried low levels of WHV DNA in serum and WHV DNA, mRNA, and cccDNA in PBMC and the lymphatic organs, while the liver was WHV negative in some of them. This was accompanied by the absence of serological markers of WHV infection, such as WHsAg, anti-WHc or anti-WHs, during the 42-month follow-up after birth. Nevertheless, WHV DNA reactive particles demonstrating biophysical properties of intact WHV virions were found in their sera, while serial liver biopsies were WHV non-reactive and showed normal histology. Healthy woodchucks inoculated with virus concentrated by ultracentrifugation from supernatants of cultured lymphoid cells and sera from these offspring developed classical serum WHsAg and anti-WHc positive acute hepatitis. These findings confirmed liver pathogenic competence of the persisting virus. Not less surprisingly, the offspring were susceptible to infection with a large dose of WHV known to cause acute hepatitis indicating that they did not establish protective immunity against reinfection (Coffin and Michalak, 1999). This study showed for the first time that hepadnaviral infection could be restricted to the immune system arguing that hepadnavirus infecting at very low copy numbers is preferentially lymphotropic.

The following study aimed to determine the dose of WHV required to establish primary occult infection in adult immunocompetent woodchucks and uncover virological characteristics and potential transmission of this form of infection (Figure 1). Healthy WHV-naïve animals were *i.v.* injected with serial 10-fold dilutions of a well-characterized WHV inoculum known to consistently cause acute hepatitis (Michalak et al., 2004). The results demonstrated that primary occult infection is triggered by *i.v.* injection of <10^3^ virions and that this infection can be reproducibly induced and serially transmitted to healthy, virus-naïve animals. The study also found that a higher *i.v.* dose (> 10^3^ virions) of the same WHV inoculum induced classical acute hepatitis that progressed to chronic hepatitis in some animals. Taken together, the findings revealed that: (1) The immune system is the primary target and can be the only site of hepadnavirus replication when virus enters the host at very low doses (< 10^3^ virions); (2) A reproducible animal model of POI was established, and (3) WHV administered *i.v.* at > 10^3^ virions invades both the immune system and the liver and causes serologically and histologically evident hepatitis. These observations have led to the concept of a liver pathogenic *vs.* a liver non-pathogenic dose of hepadnavirus.

To determine the lifetime consequences of POI, infection was established by *i.v.* injection with 10–100 WHV DNase digestion-protected virions and followed throughout the injected animals’ lifespan (Mulrooney-Cousins et al., 2014). POI was initially restricted to cells of the immune system with serum WHV DNA loads of <100–200 vge/mL and with <10^3^ vge/μg DNA in PBMC. After ∼2 years p.i., the liver became WHV positive with WHV DNA, mRNA, and cccDNA detectable at very low levels by nested PCR/NAH, although hepatic histology remained normal. However, ∼20% of the animals developed HCC. This indicated that virus persisting at very low-level retains its pro-oncogenic potency with HCC development in the absence of chronic hepatitis or other form of prolonged liver injury. Consistent with this observation was the finding of WHV DNA integration into liver as well as in lymphatic organ and PBMC genomes (Mulrooney-Cousins et al., 2014). This study highlighted the pathogenic significance of POI. Thus, serologically silent WHV infection with virus DNA detected by highly sensitive testing and restricted to the immune system eventually spreads to the liver and can cause HCC. A similar phenomenon may occur with HBV infection and be a causative factor in cryptogenic HCC. This issue would require in depth future investigation in humans with seronegative OBI (Zerbini et al., 2008; Raimondo et al., 2019). Another unresolved issue is whether newborns from asymptomatic mothers convalescent from hepatitis B could have seronegative OBI, transmit infection, and whether there are long-term consequences of such infection. Other studies identified unique characteristics of WHV-specific immune responses, indicated the absence of anti-WHV protective immunity in POI, and showed that repeated exposures to small amounts of WHV causing POI do not culminate in serologically evident infection and hepatitis (Gujar and Michalak, 2009; Gujar et al., 2013; Mulrooney-Cousins and Michalak, 2015).

## Hepadnavirus Integration Into Immune Cell and Lymphatic Organ Genomes

The finding of HBV and WHV DNA integrations into immune cell and lymphatic organ genomes provided further compelling evidence of the viruses’ lymphotropic nature (Figure 2). The presence of virus-host genomic junctions is an indicator of successful viral entry and release of DNA that is prone to fusion with cellular genome (Chauhan and Michalak, 2021). Since HBV and WHV DNA loads and replication levels are 10–100-fold lower in PBMC than hepatocytes (Korba et al., 1986; Michalak et al., 1999, 2004; Mulrooney-Cousins and Michalak, 2015), it was expected that the frequency of virus-host junctions would also be lower in immune cells and hence require more sensitive detection techniques. Nonetheless, the first indications of HBV DNA integration into circulating lymphomononuclear cell genomes were obtained using NAH analysis. Thus, integrated HBV has been reported in PBMC from patients with hepatitis B and HIV co-infection and acquired immunodeficiency syndrome, although the poorly defined patterns of HBV DNA fragments after restriction enzyme digestions were inconclusive (Pontisso et al., 1984; Laure et al., 1985). HBV integration was clearly shown in PBMC from a chimpanzee chronically infected with HBV and the fusion was confirmed by the restriction patterns of HBV-specific DNA fragments (Korba et al., 1986). For the first time, the presence of integrated HBV DNA in PBMC and the sequence of virus-cell junctions were shown by nested PCR using virus-specific biotinylated primer and an arbitrary primer specific for flanking integrated cellular DNA (Laskus et al., 1999). In 2/10 CHB patients tested, HBV DNA breaking points forming junctions were located in the virus cohesive region between direct repeat 1 (DR1) and DR2. In another study investigating PBMC using Alu element-mediated PCR (Alu-PCR), integrated HBV genome was found in 4 of 7 patients with CHB and 2 of 10 with HCV-related chronic hepatitis (Murakami et al., 2003). HBV DNA integration were also identified in nuclei of hematopoietic stem cells from bone marrow of CHB patients using fluorescence *in situ* hybridization (FISH), as previously noted (see page 6) (Ma et al., 2011). HBV DNA integration into DNA of PBMC and lymphatic tissue were also occasionally found in other studies (Umeda et al., 2005; Wang et al., 2008).

In the most recent work which applied nested Alu-PCR followed by clonal sequencing of the amplified HBV-host junctions and identification of the host integration sites (Lau et al., 2020), PBMC from a large group of patients (*n* = 53) with CHB associated or not with HCC demonstrated numerous integrations with both coding and non-coding regions of human genome. Many of the genes fused with HBV sequences were identified as those associated with the oncogenic processes in lymphoid and hepatic tumors, including oncogenes and tumor suppressor genes. Some of the integration sites were shared between PBMC and HCC. In the same study, a patient with CHB and extrahepatic lymphoid cell malignancy diagnosed as dendritic cell sarcoma (DCS) was investigated. HBV DNA, RNA, and cccDNA were detected in the patient’s PBMC and DCS tissue, as well as HBV envelope and nucleocapsid proteins were found by Western immunoblotting in this lymphoproliferative cancer. Ten unique HBV junctions with host coding genes were identified and, interestingly, HBV genotype in the tumor was distinct from that in the patient’s plasma and PBMC, i.e., genotype D versus C, respectively. This study pointed out the importance of thorough analysis of immune cells from HBV-infected patients for the presence of virus and its integration. This study provides further invaluable insights to recognition of the relationship between HBV and immune system malignancies and suspected virus-oncogenic potential (Engels et al., 2010; Li et al., 2018; Ren et al., 2018; Sinha et al., 2019).

In the woodchuck model, a study investigating lifelong outcome of POI (i.e., infection that is initially restricted to the lymphatic system), identified multiple WHV DNA-host genome junctions in PBMC and lymphatic organs, including bone marrow, spleen and lymph nodes, using inverse-PCR (invPCR) designed to specifically detect WHV X gene or WHV preS region integrations (Mulrooney-Cousins et al., 2014). WHV-host genomic fusions were found regardless of whether HCC had developed or not, and they were most often between WHV X gene and various host sequences. This last finding is consistent with the observed tendency of the HBV X gene to preferably integrate into the human hepatocyte genome (Toyoda et al., 2008; Zhang et al., 2012).

Further investigations are required to determine the consequences of HBV and WHV integration considering immune cell physiological functions and a contribution to the pro-oncogenic processes culminating in lymphoproliferative disorders, such as non-Hodgkin’s lymphoma and chronic lymphocytic leukemia (Wang et al., 2008; Nath et al., 2010; Becker et al., 2012). Recognition of the integration kinetics, diversity of the host’s sites involved, and the mechanisms mediating formation of virus-host junctions should be investigated. Similar studies have been initiated on HBV and WHV genomic fusions into hepatocyte genome and they have revealed interesting findings (Murakami et al., 2003; Chauhan et al., 2017; Tu et al., 2018). The results from these studies suggest that hepadnavirus integration into immune cell genomes may occur immediately after infection. HBV integration may involve fusions with retrotransposons and genes with translocation abilities that might spread virus sequences across the cellular genome. Additionally, the repair of cellular DNA damaged by virus-induced oxidative stress will likely be involved in the creation of virus-host DNA junctions (Murakami et al., 2003; Chauhan et al., 2019; Chauhan and Michalak, 2020).

## HBV and WHV Variants in the Immune Cell Compartment

The conversion of hepadnavirus relaxed circular DNA to cccDNA is the first step in the replication cycle, followed by transcription of cccDNA to mRNA, and then translation of the mRNA to virus proteins. Owing to an error-prone reverse transcriptase, the hepadnaviral genome is more susceptible to sequence variation than other DNA viruses. These genomic changes may occur in any virus sequence, the frequency of which is proportional to the duration of virus infection and viral fitness. The pathological implications of these mutations are unclear but recent data suggest tendency toward compartmentalization of HBV variants with possible autonomous evolution of variants in the immune cell compartment.

In this regard, a number of studies reported: (1) Persistence of HBV in PBMC without coinciding liver infection, as in liver transplant recipients (Féray et al., 1990; Repp et al., 1991); (2) Persistence of unique HBV variants in PBMC were the source cause of reinfection of the new liver (Brind et al., 1997), and (3) occurrence of different genotypes of HBV in PBMC *vs.* liver in the same transplant patient (Tai et al., 2001). Beyond the liver transplant cases, support for the site-specific compartmentalization came from demonstration of two different HBV genotypes in PBMC in the absence of HBV DNA in serum among family members with OBI (Datta et al., 2009). The genotype A with an immune escape mutation G145R within the S gene occurred in all members of this family, while genotype D in PBMC of some members only. The authors suggested different infection routes for acquisition of these two distinct genotypes by PBMC. It was also shown that sub-genotype Ae/A2 with G145R mutation was uniquely restricted to PBMC in unrelated individuals whose sera carried different HBV sub-genotypes but not Ae/A2 sub-genotype with the G145R mutation (Chakravarty et al., 2002).

In other studies, genetic variability of HBV in PBMC and liver and plasma samples acquired in parallel was examined. Studies of patients after liver transplant due to HBV infection-related liver disease showed that despite prolonged anti-HBV nucleos(t)ide analog therapy and hepatitis B immune globin (HBIG) administration, resulting in undetectable serum HBsAg and HBV DNA, HBV replication continued after transplant in both liver and PBMC. Furthermore, while the liver carried predominantly antiviral drug-resistant variants, PBMC harbored wild-type HBV sequences (Coffin et al., 2011b). This not only indicated that HBV persists and the need for continuation of anti-HBV therapy after liver transplant, but implied the compartmentalization of drug-resistant *vs.* wild-type virus populations in the liver and PBMC, respectively. Other data suggested that HBV might persist and evolve differently in PBMC than in the liver due to a different cellular microenvironment (Coffin et al., 2011a). In another study, immune escape HBV mutations were more frequently detected in PBMC than in plasma of patients with active CHB and among 22 patients studied, only three had HBV of the same genotype in both plasma and PBMC (Coffin et al., 2015). The finding of a divergent HBV genotype in PBMC was not unique to this study, particularly from the time when HBV residing in the PBMC or lymphatic tissue became more systematically sequenced and compared to that in serum and liver tissue (Datta et al., 2009; Coffin et al., 2011a, 2015; Cassini et al., 2013; Lee et al., 2015; Gao et al., 2017; Lau et al., 2018). This is well exemplified by analysis of CHB patients in a recent study in which the predominance of HBV genotype C, which is considered more liver oncogenic, and single nucleotide polymorphisms (SNPs) associated with HCC were found in PBMC when compared to plasma and liver tissue (Lau et al., 2020). The HBV genotype and the SNP profiles also were significantly different among the PBMC, plasma and extrahepatic lymphoid tumor from a patient with CHB and DCS examined in the same study (Lau et al., 2020). Collectively, the data available strongly support the notion that immune cells constitute an independent reservoir of HBV persistence in which genetic evolution of virus differs from that in the liver. They also indicate that this compartment can support propagation of HBV in the context of otherwise successful antiviral therapy as defined by sustained seronegativity or by considerable suppression in serum levels of HBV DNA and HBsAg.

Using the woodchuck HBV model to determine whether infection of immune cells is a property of wild-type WHV or a particular variant predisposed to infect these cells, WHV derived from cultured splenocytes of an animal with chronic hepatitis, which carried identical WHV sequence in serum, liver and the immune system, was serially passaged in cultured virus-naïve splenocytes and WCM-260 hepatocyte line (Mulrooney-Cousins and Michalak, 2008). It was assumed that the repeated passage of such virus within lymphoid cells should enrich a lymphotropic variant. However, the passage for up to 13 times in both cell types did not result in the appearance of cell type-specific WHV variants, as determined by the clonal sequence analysis of virus envelope, core and X gene sequences. Furthermore, WHV passaged in both cell types remained infectious to woodchucks and the infection profiles in these animals was related to the virus dose but not cell origin. The virus also retained its initial wild-type sequence in the animals infected. These data confirmed that lymphotropic WHV infects both immune cells and hepatocytes, can be serially transmitted in both cell types, and remains infectious and pathogenic to liver in susceptible animals. Taken together, the study affirmed that lymphotropism is a natural property of wild-type WHV and is not a result of infection with a uniquely lymphotropic variant.

## Discussion

The accumulated evidence indicate that the ability to infect cells of the immune system is a natural propensity of HBV and its close relative WHV. They show that the infected immune cells can be commonly found in both serologically evident, symptomatic and silent (occult), asymptomatic infections with these viruses when sensitive investigative methods are applied. Since the amounts of HBV and WHV are ∼10 to 100-fold lower in immune cells than in serum or hepatocytes their detection is challenging by standard approaches and therefore testing of larger sample amounts and serial samples from a given infected host could augment detection frequency (Michalak et al., 2007). The pathogenic and clinical implications of HBV lymphotropism are not yet fully recognized and are still debated. However, the following aspects of the HBV ability to infect cells of the immune system have been recognized and are important: (1) The site of long-term virus persistence; (2) A reservoir of replicating virus available for infection of HBV-naïve liver tissue, as in liver transplants, vertical transmission of infection *in utero*, and possible reactivation of asymptomatic infection; (3) The site of virus presence and replication despite otherwise successful antiviral therapy; (4) The site of autonomous, liver-independent evolution of virus variants; (5) The place of virus genome integration possibly underlying lymphatic system disorders, and (6) An easily accessible source of clinical and research material for diagnostic and treatment evaluation purposes, and assessment of the efficacy of novel anti-HBV approaches. Based on the findings summarized in the previous sections, the issues mentioned above will be discussed and supplemented with additional information (Figure 3).

### HBV Persistence

The analysis of PBMC and their cell subsets from patients with advanced CHB, regardless of HCC status, indicated that HBV persists until the-end-stage liver disease. The presence of HBV and its replicative intermediates, including cccDNA, did not significantly decline in PBMC with advancement of CHB and development of HCC, as a recent study showed (Lau et al., 2020). HBV and its replication also remain detectable in PBMC during OBI, that may also culminate in HCC (Michalak et al., 1994; Raimondo et al., 2008; Joshi and Coffin, 2018; Mak et al., 2020). The enhanced expression of HBV genome and its mRNA and cccDNA after *ex vivo* treatment of PBMC with mitogens offers an opportunity to detect virus or confirm its presence in situations of very low viral loads or the apparent absence of virus in these cells (Yan et al., 2016; Lau et al., 2020). However, data from studies on HCV that also replicates and persists in various immune cell types, indicate that the mitogen treatment of individual PBMC cell subsets may facilitate virus detection even when mitogen-treated total PBMC appears to be virus non-reactive (Pham et al., 2005, 2008). In the case of HCV, this suggested preferential or exclusive infection of a particular cell type (Pham et al., 2008). The same might apply to PBMC infected with HBV. The woodchuck-WHV model offers ideal conditions for infection with desired virus doses and collection of serial liver and PBMC samples during the lifespan, as well as tissues at autopsy. Thus, lifelong persistence of WHV in PBMC and lymphatic organs was unequivocally demonstrated, even when infection was induced with doses as low as 10 virions (Mulrooney-Cousins et al., 2014; Mulrooney-Cousins and Michalak, 2015). Treatment of immune cells with mitogens was also applied to detect WHV residing in immune cells at levels below detection limits of the nested-PCR/NAH assays or when immune cell-derived virus was required for proof-of-concept *in vivo* or *in vitro* infection experiments (Coffin and Michalak, 1999; Michalak et al., 1999, 2004; Lew and Michalak, 2001). Further, the woodchuck model of POI showed the close relationship between hepadnaviral lymphotropism and persistence, highlighting that WHV can persist solely in the immune cell compartment without involving the liver, and that this state can last for many months after infection with small amounts of virus (Coffin and Michalak, 1999; Mulrooney-Cousins et al., 2014). Further, it is expected that long-term presence and propagation of HBV in the immune cell compartment may exert adverse effect on immune cell functions and hence facilitate viral persistence. There are very limited data in this regard, as already mentioned in this review. They postulate that HBV infection of hematopoietic stem cells could suppress hematopoiesis and result in the generation of defective T cells which proliferative capacity and production of cytokines are potentially becoming compromised (Steinberg et al., 1990; Ma et al., 2011). This area requires more investigation before drawing conclusions.

### Reservoir of Infectious HBV

Hepatitis B virus residing in PBMC can infect HBV-naïve liver transplants, as explicitly shown by the appearance of PBMC-unique variants in livers of the transplant recipients (Cassini et al., 2013; Coffin et al., 2015; Lee et al., 2015). In this regard, infection of livers with WHV produced by cultured, naturally infected PBMC and splenocytes resulted in development of acute and chronic hepatitis followed by HCC in the woodchuck infection model (Michalak et al., 1999, 2004; Lew and Michalak, 2001; Mulrooney-Cousins and Michalak, 2015). It has also been shown that HBV-infected PBMC play a role in transmission of virus *in utero* from infected mother to fetus by crossing the placenta (Bai et al., 2011; Shao et al., 2013; Xu et al., 2015). This remains in agreement with reports indicating the existence of the two- way cell trafficking between mother and fetus across the placental barrier (Lo et al., 1996, 2000). In one of the studies, 83.8% of the HBV DNA positive maternal PBMC have passed through the barrier and entered fetal circulation (Xu et al., 2015). Furthermore, PBMC traffic from infected mother to fetus was associated with a significantly greater risk of HBV infection in infants whose PBMC were HBV DNA positive. These data indicate that HBV-infected maternal PBMC can contribute to infection of newborns. This represents an alternative route to HBV mother-to-child transmission, which was previously thought to occur at the time of birth or during the perinatal period due to infant contact with infected maternal body fluids. Nonetheless, follow-up studies are required to recognize the pathological consequences for the infants infected *via* maternal PBMC, particularly regarding a possible predisposition to immunoproliferative diseases later in life. There is limited parallel experimental data from the woodchuck-WHV model. However, mothers with resolved acute hepatitis carrying small amounts of WHV in serum PBMC and liver, and in the presence of anti-WHs antibodies transmitted infection to their offspring in which virus was detectable in serum, PBMC and lymphatic organs but not in the liver (Coffin and Michalak, 1999). It remains unknown if infection was transmitted by plasma or PBMC, however, since it occurred in the presence of maternal anti-WHs antibodies this argues that virus transfer was *via* infected circulating immune cells.

### HBV Variants and Their Compartmental Evolution

As cumulative data indicate, HBV can propagate within immune cells in the context of otherwise successful antiviral therapy, and evolve into distinct variants compared to virus in the liver. HBV variants occurring in serum are assumed to mainly originate from the infected liver and to a lesser degree from immune cells or other possible extrahepatic locations. Thus, analysis of variants in serum samples may be a practical alternative to compare hepatic *vs.* extrahepatic evolution, if liver samples are unavailable. The exception is POI where infection is limited to the immune system and does not initially involve the liver. The strongest evidence of distinct compartmentalization of HBV variants is detection of different virus genotypes or sub-genotypes in PBMC *vs.* plasma and/or liver (Datta et al., 2009; Coffin et al., 2015; Gao et al., 2017; Lau et al., 2020). Furthermore, HBV residing in PBMC was found to replicate despite prolonged NA therapy. This *per se* is not surprising since NA treatment suppresses HBV levels but cannot eliminate cccDNA and eradicate the virus. Nonetheless, it was observed that while HBV DNA decreased to very low levels in plasma following NA therapy, no significant changes in the levels of HBV DNA and cccDNA occurred in PBMC (Gao et al., 2017). It was also observed that wild-type HBV sequences predominate in PBMC during NA therapy, although the drug resistant variants can be also detected (Coffin et al., 2011a). Further, the HBV genotype can differ before and after the NA treatment in different compartments, i.e., liver, PBMC and plasma, and can be switched between PBMC and plasma (Gao et al., 2017). The development of compartment-specific variants might be due to variances in the pre-genomic RNA secondary structure (Datta et al., 2009). Other possible contributors leading to autonomous evolution of variants in the immune cell compartment include: (1) Differences in the microenvironment within immune cells and hepatocytes; (2) Differences in immune pressure on virus propagating within immunologically privileged sites, such as immune cells and hepatocytes; (3) Diverse kinetics of virus replication with either higher or lower rates in immune cells *vs.* hepatocytes, and (4) Different pharmacokinetics with respect to penetration of antiviral agents into immune cells and hepatocytes (Figure 3).

### HBV Integration

Hepatitis B virus integration into the PBMC genome was identified in early investigations on hepadnaviruses, however host sites involved in formation of virus-host junctions was recently recognized (Lau et al., 2020). Detailed analysis of HBV genomic fusions in PBMC from patients with CHB revealed numerous virus DNA merges with host genes, including genes known to be involved in pro-oncogenic processes. The same study also demonstrated for the first time, HBV expression, replication and integration within an extrahepatic lymphoproliferative tumor in one CHB patient (Lau et al., 2020). There are many reports showing an increased risk of development of non-Hodgkin lymphoma, particularly diffuse large B cell lymphoma in the course of chronic HBV infection (Nath et al., 2010; Becker et al., 2012; Li et al., 2018; Su et al., 2019; Zhou et al., 2019). Therefore, it may be valuable to perform additional similarly detailed investigations of immunoproliferative neoplasms occurring in patients with CHB or individuals with past exposure and silent (occult) HBV infection. The existence of numerous HBV-host genomic fusions in PBMC and other immune cells of chronically infected individuals may parallel effects seen in HBV-infected hepatocytes in which modification of individual gene expression causes overall destabilization of the cellular genome (Wollersheim et al., 1988; Saigo et al., 2008; Sung et al., 2012; Levrero and Zucman-Rossi, 2016). These effects could have a strong pro-oncogenic potency resulting in malignant transformation. Furthermore, next generation sequencing of HBV-infected PBMC from CHB patients showed the presence of genetic variants associated with enhanced risk of HCC development (i.e., A1762T/G1764A) (Lau et al., 2020). In another study, OBI was identified in the majority (67.5%) of patients with diffuse large B cell lymphoma and HCC-associated HBV variants were identified in plasma, PBMC and lymphoid tumor tissue of these patients (Sinha et al., 2019). These data overall support the longstanding concept regarding the oncogenic role of HBV lymphotropism in the pathogenesis of immune cell malignancies.

### Diagnostic and Research Relevance of HBV Lymphotropism

The easy access to PBMC allows serial sampling and investigation of possible scenarios to enhance clinical diagnosis and monitoring of HBV, including detection and assessment of replication status during progression and resolution of hepatitis B. PBMC also provide valuable material for evaluating the efficacy of novel antiviral therapies. This may allow future comprehensive assessment of the status of HBV infection, including virus clearance due to treatment using advanced molecular techniques, i.e., analysis of PBMC and HBV transcriptomes and single-immune cell RNA-sequencing. However, there remains an ongoing need to evaluate HBV in the liver. Although HBV DNA loads and replication levels are generally lower in PBMC than in the liver, viral kinetics in PBMC follow well those in hepatic tissue, based on data from a few comparative studies in HBV-infected humans (Coffin et al., 2011a,b; Gao et al., 2017; Lau et al., 2020). These kinetics have been well recognized in the woodchuck model of HBV infection (Michalak et al., 1999, 2004; Mulrooney-Cousins et al., 2014). In cases with very low HBV loads or apparently undetectable HBV in serum or plasma, HBV is still often detected in the PBMC compartment using assays of the same sensitivity. In addition, *ex vivo* stimulation of PBMC with individual mitogens or their cocktails, which augments virus genome expression and replication, offers a unique opportunity for assessing elimination of virus in cases where liver biopsy is virus non-reactive. As indicated previously, examination of PBMC provides an advantage for OBI detection, particularly its seronegative form, i.e., HBsAg, anti-HBc, and anti-HBs negative (Raimondo et al., 2019), as it was documented for POI in the woodchuck-WHV infection model (Coffin and Michalak, 1999; Gujar and Michalak, 2009; Mulrooney-Cousins et al., 2014; Mulrooney-Cousins and Michalak, 2015). The sensitivity of molecular assays currently used in clinics to detect HBV DNA could be improved by using PBMC, in addition to serum or plasma, as test material. Further, there are several non-commercial tests for identification of HBV cccDNA or mRNA in clinical samples. The use of PBMC may be of benefit for occult HBV diagnosis and evaluation of antiviral treatment, especially given the convenient, less invasive method of sample collection. It might be argued that evaluation of PBMC could be challenging for clinical laboratories, however, there is significant progress in simplifying PBMC isolation without jeopardizing cell viability and nucleic acid integrity [e.g., by using Vacutainer cell preparation tubes (Corkum et al., 2015)] and in automation of nucleic acid isolation and PCR. Moreover, HBV eradication and prevention of virus-induced pathogenic and oncogenic consequences is not possible without a sterilizing cure, even for an asymptomatic infection. Therefore, evaluation of the efficacy of antiviral therapy should include immune cells, as represented by PBMC.

Despite evidence from studies on other non-cytopathic viruses establishing persistent infections, research on HBV lymphotropism has been hampered by difficulties in virus detection in the immune cell compartment as well as by an underappreciation for the role of lymphotropism in hepadnaviral biology and pathogenicity. The authors are hopeful that this review emphasizes the importance of this subject and will encourage further definitive studies.

## Author Contributions

TM conceived, designed, and wrote the draft. CC edited the manuscript and provided the additional information. PM-C provided the complementary data and reviewed the manuscript. All authors contributed to the article and approved the submitted version.

## Conflict of Interest

The authors declare that the research was conducted in the absence of any commercial or financial relationships that could be construed as a potential conflict of interest.

## Publisher’s Note

All claims expressed in this article are solely those of the authors and do not necessarily represent those of their affiliated organizations, or those of the publisher, the editors and the reviewers. Any product that may be evaluated in this article, or claim that may be made by its manufacturer, is not guaranteed or endorsed by the publisher.
